# Knowledge, perceptions and myths regarding infertility among selected adult population in Pakistan: a cross-sectional study

**DOI:** 10.1186/1471-2458-11-760

**Published:** 2011-10-04

**Authors:** Sumera Ali, Raafay Sophie, Ayesha M Imam, Faisal I Khan, Syed F Ali, Annum Shaikh, Syed Farid-ul-Hasnain

**Affiliations:** 1 Medical College, Aga Khan University Stadium Road, Karachi, Pakistan, PO Box 3500; 2 University of Mary Washington, 1301 College Avenue, Fredericksburg, VA 22401, USA; 3 Department of Community Health Sciences, Aga Khan University Stadium Road, Karachi, Pakistan, PO Box 3500

**Keywords:** Knowledge, attitudes, perception, myths, infertility, infertility treatment, Pakistan

## Abstract

**Background:**

The reported prevalence of infertility in Pakistan is approximately 22% with 4% primary and 18% secondary infertility. Infertility is not only a medical but also a social problem in our society as cultural customs and perceived religious dictums may equate infertility with failure on a personal, interpersonal, or social level. It is imperative that people have adequate knowledge about infertility so couples can seek timely medical care and misconceptions can be rectified.

We aim to assess the knowledge, perception and myths regarding infertility and suggest ways to improve it.

**Methods:**

A cross-sectional survey was carried out by interviewing a sample of 447 adults who were accompanying the patients at two tertiary care hospitals in Karachi, Pakistan. They were interviewed one-on-one with the help of a pretested questionnaire drafted by the team after a thorough literature review and in consultation with infertility specialists.

**Results:**

The correct knowledge of infertility was found to be limited amongst the participants. Only 25% correctly identified when infertility is pathological and only 46% knew about the fertile period in women's cycle. People are misinformed that use of IUCD (53%) and OCPs (61%) may cause infertility. Beliefs in evil forces and supernatural powers as a cause of infertility are still prevalent especially amongst people with lower level of education. Seeking alternative treatment for infertility remains a popular option for 28% of the participant as a primary preference and 75% as a secondary preference. IVF remains an unfamiliar (78%) and an unacceptable option (55%).

**Conclusions:**

Knowledge about infertility is limited in the population and a lot of misconceptions and myths are prevalent in the society. Alternative medicine is a popular option for seeking infertility treatment. The cultural and religious perspective about assisted reproductive technologies is unclear, which has resulted in its reduced acceptability.

## Background

Infertility is a disease of the reproductive system which affects both men and women with almost equal frequency [1]. While there is no universal definition of infertility, a couple is generally considered clinically infertile when pregnancy has not occurred after at least twelve months of regular unprotected sexual activity [2]. In 90% of the cases the cause is identifiable and in 50% of the cases appropriate therapy will result in pregnancy [1].

Infertility is a global phenomenon that affects between 60 million and 168 million people worldwide [3]. The majority of those who suffer live in the developing world. WHO-DHS Comparative Report in 2004 states that more than 186 million ever-married women in developing countries (excluding China) were infertile because of primary or secondary infertility [4]. This number represents more than one in four ever-married women of reproductive age in these countries. The prevalence of infertility in Pakistan is 21.9% where, primary infertility is 3.9% and secondary infertility is 18.0% [5].

Infertility is a source of distress for couples as societal norms and perceived religious dictums may equate infertility with failure on a personal, interpersonal, emotional or social level. Women bear the brunt of these societal perceptions in most of the cases. Psychologically, the infertile woman exhibits significantly higher psychopathology in the form of tension, hostility, anxiety, depression, self-blame and suicidal ideation [6]. In Latin America, strong social stigma attached to infertility and machismo cause women to blame themselves for infertility [7] while in Mozambique, infertile women are excluded from certain social activities and traditional ceremonies [8]. Social stigma regarding infertility is especially common across South Asia. For e.g. in Andhra Pradesh, India 70% of women experiencing infertility reported being punished with physical violence for their failure [9]. Women are verbally or physically abused in their own homes, deprived of their inheritance, sent back to their parents, ostracized, looked down upon by society, or even have their marriage dissolved or terminated if they are unable to conceive [10-12].

In Pakistan, bearing progeny is regarded as part and parcel of a stable marital nexus. Children, particularly sons, are regarded as a source of income and security in old age. A study conducted in Karachi on the psycho-social consequences of secondary fertility [5] revealed that more than two thirds (67.7%) of women, who were unable to give live births or give birth to sons had marital conflicts. These women had been threatened with divorce (20%), husband's remarrying (38%) or were being forced to return to their parent's home (26%) by their in-laws or husbands. They also reported that they were being physically and verbally abused by their husbands and in-laws leading to severe mental stress. It is a common view in Pakistani culture that infertility is not a disorder and being blessed with children is only by God's Will. If people don't recognize infertility as a disorder, it may prevent them from seeking timely medical care for the correctable causes of infertility. However, the prevalence and impact of this belief remains undetermined in our population.

Knowledge about infertility is inadequate in many parts of the world. A global survey of almost 17,500 women (mostly of childbearing age) from 10 countries revealed that knowledge regarding fertility and biology of reproduction was poor [13]. Many women have little awareness of the period of the month in which they are most fertile and when to seek treatment [14,15] In addition to the low level of knowledge, there are a number of misconceptions regarding infertility all over the world. In Tanzania for instance, evil forces are often thought to be the cause of infertility [16]. These misconceptions eventually lead to practices ranging from the absurd post-coital exercise of standing on one's head [17] to the unpleasant and dangerous traditional remedies of eating feces and inducing vomiting in Tanzania.

Research exploring the knowledge, behaviors, perceptions and practices regarding infertility or certain treatment options have been carried out in Nigeria [18], Iran [2], Wales [19], South Africa [20] and other countries but very limited data is available from the population in Pakistan despite such a high prevalence. With this study we hope to achieve a better understanding of the level of awareness and misconceptions of infertility in Pakistan which has not yet been explored.

## Methods

### Site and Study Design

A cross-sectional survey was conducted on a conveniently selected adult population, recruited from the outpatient centers of two tertiary care hospitals in Karachi, Pakistan (Aga Khan University Hospital and Liaquat National Hospital). These two hospitals were chosen because they are the largest private hospitals in the city and people from all socio-economic and ethnic backgrounds visit these centers. An outpatient center was chosen at each hospital, which had designated waiting areas for patients and people accompanying them for their doctor's visit.

### Recruitment and Interview

The patient's attendants, who were defined as people accompanying the patient, were randomly approached by the study team members in the waiting lounges. Individuals working at the same institution and patients were excluded from the study. After taking a written informed consent, participants were interviewed one-on-one at the designated locations, where privacy was ensured. Each interview was conducted by a team member who had undergone previous training. The interview was conducted in the native language of Urdu, using a structured, pre-tested questionnaire and lasted between 12-15 minutes.

### Sample Size

Using the formula (z^2 ^× p × q)/E^2 ^a sample size of 385 was calculated with a confidence interval of 95% and precision of 5%. This sample size was inflated by 20% to account for the non-responders. Therefore, the adjusted sample size was approximately 460.

### Questionnaire

The questionnaire was initially designed by the research team on the basis of previously published literature on infertility [15,19,21] and later two infertility specialists were consulted for their opinion. It was originally designed in English and then translated into Urdu by a professional translator. It was thoroughly discussed amongst the study team and external experts to reduce any bias and to standardize the terms. One of the terms that raised discussion amongst the panel was "test tube baby". To our knowledge and from the opinion of the experts, "test tube baby" is used as it is in the native dialect to refer to assisted reproductive technologies (ART) and there is no exact translation available for this term in Urdu. It is also a commonly used phrase in the media and also by the infertility specialists when counseling patients.

The questionnaire was divided into various subsections, the first assessed knowledge of infertility and the next evaluated people's attitude towards infertility. The final section inquired about their perceptions of how infertility affects marital outcomes and explored prevailing myths about infertility in the context of Pakistani culture and society.

The prevailing myths that were included in the questionnaire were identified during the pilot phase of the study, when people were asked open ended question about what they thought were the prevailing myths in the culture. The common responses were then included in the final phase of the interviews.

### Statistical Analysis

Data was coded and entered using EpiData v3.2. Database files were then exported to SPSS v16.0 to be analyzed. Associations were assessed using Chi-square test; a p-value of ≤ 0.05 was taken as significant.

### Ethical Considerations

The research protocol was reviewed and approved by the Ethics Review Committee at the Aga Khan University. All subjects had the right to withdraw from the study anytime they wished without giving any explanation. The questionnaire was anonymous and ensured confidentiality of the study participants. Written informed consent was taken from each participant prior to the interview.

## Results

### Socio-Demographic Characteristics of the Sample

A total of 460 individuals were approached to participate in this survey. Four hundred and forty seven individuals agreed to participate; therefore, the response rate in the study was 97.2%. Respondent's ages ranged from 18 to 75 years old with a mean age of 36 ± 13 years. There were a total of 201(45%) males and 246(55%) females in this study. Participants were classified according to their educational status into level I (≤ 5 years of formal education), level II (6-10 years) and level III (> 10 years). Forty six percent of the sample belonged to level III. Almost all the participants (98%) were Muslims. The socio-demographic characteristics of the study population are summarized in table 1.

**Table 1:** Socio-demographic profile of the individuals interviewed.

		Total	
		
Socio-demographic variable		N	%
Age	18-26 27-32 33-44 45-75	119 107 110 111	26 24 25 25

Sex	Male Female	201 246	45 55

Education Level	I II III	57 184 206	13 41 46

Occupation	HCP NHCP HW Student	48 184 165 49	11 41 37 11

Marital Status	Unmarried Married	117 330	26 74

### Knowledge About Infertility

The results showed that only 25.0% of the participants correctly recognized that infertility is diagnosed usually after one to two years of regular unprotected sex while most of them believed that it is either less than twelve months or more than 3 years.

The next question was about whom they thought was the cause of infertility most of time and 40% of them correctly identified that both male and female are equally responsible. Seventy percent of the participants were aware that there is a fertile period during a female's menstrual cycle. However when they were asked to identify that period from the choices given (right after menses, mid cycle or just before the beginning of menses), only 46% of them correctly identified mid-cycle.

Participants were further asked to identify the major causes of infertility from the list provided, which consisted of both correct and incorrect responses (Table 2). More than 70% of the participants correctly identified irregularity of menses, blocked tubes and genital tract infections as a cause of infertility. Many interesting responses were observed: 76% of them did not think smoking is a cause of infertility, and more than 50% of participants thought that previous use of oral contraceptive pill (OCP) and intra-uterine contraception device (IUCD), leads to infertility.

**Table 2:** Knowledge of the respondents regarding the factors which influence infertility and common misconceptions.

	Causes of Infertility	N	Yes n (%)
1	Abnormal menses (ovulatory factors)	410	350 (85%)

2	Blocked tubes	408	383 (94%)

3	History of Infections of genital tract in females	402	296(74%)

4	History of infections of genital tract in males	392	283(72%)

5	Smoking	420	102(24%)

6	Previous use of contraceptive pills by female	400	244(61%)

7	Previous use of Intrauterine device by female	376	200(53%)

8	*Jinns*/Supernatural causes	441	134(30%)

9	Black magic	455	175(38%)

10	Regular Exercise	433	57(13%)

11	Psychological stress	442	289(65%)

12	Being obese	435	253(58%)

The knowledge of common investigations required for infertility was also assessed and most of them correctly identified the tests that are required. The only exception was the hysterosalpingogram or 'tubes X-ray', which 44% of the people did not know. When asked who should be investigated first, 55% answered both at the same time while rest of them either chose male or a female.

On comparative analysis, a general trend in knowledge was observed; females had significantly better knowledge than males on most of the questions (p-value < 0.01). People with higher education had better knowledge as compared to the less educated ones as expected (p-value < 0.05).

### Attitude Towards Infertility (Table 3)

It was interesting to find that 45% of people did not want to label infertility as a 'disease'. More females (56%) were of the opinion that infertility is not a disease as compared to males (31%). Even though they had differing views about whether to call it a disease or not, 94% of them believed that couple should seek treatment for it. Ninety seven percent of those with a higher level of education said there was medical treatment available compared to 87% of those with no formal or primary education (p < 0.05).

**Table 3:** Attitude of the respondents regarding infertility.

Questions pertaining to attitudes	Response	Total	
		
		*n*	%
Do you think infertility is a disease?	Yes No	244 198	55 45

Do you think infertility should be treated medically?	Yes No	405 28	94 06

Who do you think should be investigated first?	Husband Wife Both	85 107 239	20 25 55

Do you think that if a couple conceives once they might have problems conceiving again?	Yes No	323 115	74 26

Whom would you go for treatment? Primary preference?	Gynecologist Others(Hakeem, Faith healers, homeopathic)	323 122	72% 28%

Secondary preference?	Gynecologist Others(Hakeem, Faith healers, homeopathic)	112 335	25% 75%

The majority chose to initially consult a gynecologist for the treatment but if unsuccessful, 75% would alternative treatments from *Hakeems*, faith healers and homeopathic practitioners. Chi-Square was computed to find the association between the education level and their preference for treatment. Predictably, only 43% of the people with education level I preferred gynecologist as a primary preference in comparison to 80% with higher levels of education (p < .05).

### Perception and Myth Towards Infertility (Table 4)

Participants were also asked about their views on infertility and marital outcomes. The majority of participants did not think male or female infertility were grounds for divorce (Ninety one percent of them did not consider female infertility as grounds for divorce, as opposed to 59% who did not consider male infertility as grounds for divorce). However, 57% believed that female infertility is a valid reason for a man to have a second marriage. Out of the people who were in favor of the second marriage, 70% were male (p < 0.05). On inquiring who they thought was being blamed for infertility in the society, most of the respondents replied that it is usually the woman (86%).

**Table 4:** Perceptions about marital outcomes and options for infertile couple

		Total	
		
*Questions pertaining to Perception about marital outcomes*	*Response*	*n*	%
If a female cannot have a baby, do you think this is grounds for divorce?	Yes No	37 406	08 92

If a female cannot have children, do you think this is a valid reason for the man to have a second marriage?	Yes No	254 189	57 43

If a male cannot have children, do you think this is grounds for divorce?	Yes No	179 260	41 59

If a couple cannot have a child, do you think they should adopt?	Yes No	406 37	92 08

Who is being blamed for infertility in the society?	Husband Wife Both Neither	17 382 23 24	04 86 05 05

Do you think it is socially acceptable to have a test-tube baby?	Yes No	140 174	45 55

Do you think fertility drugs are socially acceptable?	Yes No	139 9	94 06

Participants were further asked about the social acceptability of various options available for infertile couples. Out of the people who were aware of fertility drugs for treatment (11% of total participants), 94% considered it to be acceptable. However, having a test-tube baby was not socially acceptable to the majority (55%) of patients who knew about it (22% of total participants). People were quite positive about the option of adopting a child and 92% agreed upon adoption as an option for infertile couples.

There is a prevalent belief in the society that infertility can be caused by supernatural causes like *jinn *(evil spirits) and black magic. To know about people's views on these beliefs we included these two options in the list of causes of infertility. We found that 30% of the people we interviewed believed *jinns *to be a cause of infertility while almost 40% believed that black magic could be a cause of infertility. A significant difference was found between people's perception regarding supernatural powers within different educational levels (p < 0.05). Most of the people (70%) belonging to the education level I, took *jinns *to be a cause compared to 20% from level III. Similarly 75% of group with education level I believed black magic to be a cause compared to 30% of those in level III.

## Discussion

The results of this study indicate that knowledge about infertility is limited in the study population. For instance, more than half of the participants were misinformed that use of IUCD and OCPs may lead to infertility. The most interesting finding of this study was that the majority of individuals would prefer alternative treatment options, if unsuccessful with the allopathic medicine. Also, half of the participants considered a "test tube baby" an unacceptable option, despite its acceptability by religious dictums. Another significant finding regarding perception of infertility was subjects' beliefs in the evil forces and supernatural powers as a cause of infertility, which correlated with their education level.

The inadequacy of knowledge about infertility was clearly demonstrated through this study. This lack of knowledge explains why such a strong stigma is attached to infertility in the society. The results of this study are similar to that of a large global survey conducted during the World Fertility Awareness Month (2006) on approximately 17,500 individuals, which revealed that the knowledge regarding fertility and biology of reproduction was lacking throughout the world [22]. The limited knowledge was further confirmed upon discovering that merely one-fourth of the participants knew how infertility is diagnosed after at least two years of regular unprotected sex. This may subsequently determine when the couple will start seeking treatment, which should be neither premature nor delayed. It is also important for the elderly in the society to have some awareness about infertility. In that way, they will not pressurize young newlyweds, if they are unable to conceive right after the marriage, which is a common expectation in the joint family structure in Pakistan.

In addition to proper knowledge about infertility, it is also crucial to know the most fertile period for a woman when she is trying to conceive. One of the surprising results found in this study was that only 46% of the participants correctly identified mid-cycle as the most fertile period during the female's menstrual cycle. The lack of accurate information in this case may lead to improper timings of sexual intercourse, thus possibly delaying the pregnancy.

While testing the subjects' knowledge, we also assessed what they considered to be the causes of infertility. Although it is not important that the general public know all the causes, it is important for them to know about acquired and potentially preventable causes of infertility such as sexually transmitted diseases. The participants in this study correctly identified most of the causes of infertility but also incorrectly highlighted factors that do not cause infertility such as use of IUCD and OCP. This may lead to underutilization of contraception for incorrect fear that the method will cause sterility and contribute to the already high rates of parity in the developing countries. These results are supported by another study conducted by Bunting [19].

While the limited knowledge about infertility was an important discovery, the most interesting finding was about people's attitude towards infertility. Though majority of the participants believe that the couple should seek treatment, not all of them would seek allopathic practitioners and instead would seek alternative treatments. In this study, it was noted that alternative medicine options, such as visiting *Hakeems *(15%) and *pirs *(faith healers) (13%) were considered acceptable. This reflects prevalence of the strong belief that all ailments cannot be cured by medical science.

Another interesting finding of this study was the correlation between beliefs in evil forces or supernatural powers as causes of infertility and the education level of the subjects. Some respondents (30%) believed that if the female is not able to conceive, she may be possessed by an evil spirit. The less educated participants were more likely to attribute the causes of infertility to an evil force or supernatural power, outside human control. In fact, these findings are confirmed by another study which was conducted amongst the Kuwati infertile women. It was discovered in that study that the uneducated group attributed the causes of their infertility to supernatural causes such as evil spirits, witchcraft and God's retribution, while the educated group held nutrition, marital and psychosexual factors responsible for their infertility [13].

In order to consider all these perceptions in a proper light, it is important to consider the societal norms and culture of Pakistan. In this part of the world, it is usually the woman who is being blamed for infertility as a reaction to a couple without children, which was confirmed through the study. Another study conducted by *Sami et al. *in Pakistan revealed that 69% of the secondarily infertile women reported being blamed for infertility often by in-laws, followed by husbands (38%) [23]. She further reported that one third of the women were blamed to be unlucky not only to the husbands but also to the entire family. The placement of unnecessary blame on a woman can potentially affect her self-esteem and become socially crippling for her.

As a result of the improper blame placed on a woman, infertility can be a common cause of marital discordance between couples. This issue was addressed by asking the subjects their views on divorce and husbands' remarrying in case of infertility. It was disturbing to discover that people believe husbands should be allowed to remarry and have a second wife in case he is not able to conceive with the previous wife. According to Islam, the religion of majority of Pakistan, infertility is not a ground for divorce for either the male or the female. Yet this study showed that people were still in favor of divorce. This implies disconnection between people's religious beliefs and their beliefs about infertility.

Despite the disconnection, religion and customs continue to play a major role in the practices related to infertility. Hence, it makes sense that knowledge about treatment option for infertility, such as IVF, is very low because it is an advanced option with limited availability in Pakistan. Amongst the respondents who knew about it, 55% considered it unacceptable because of beliefs that it's not allowed in Islam or that the procedure may use foreign egg or sperm. This reveals that even the small group of people that knew about IVF is misinformed. Individuals may not be aware that IVF and similar technologies are permissible in Islam as long as they do not involve any form of third-party donation [24,25]. While IVF has been explored in other Islamic countries such as Iran, this issue needs further exploration and active debate in Pakistan.

## Limitations

This was a study conducted on healthy individuals and respondents' own history of infertility and use or IVF was not collected. It would have been interesting to see the association of personal history of infertility with their attitude towards marital discordance and their use of alternative treatment options. Furthermore, the personal history of IVF may have affected the results on knowledge of infertility treatment options. During the survey, the distinction between primary and secondary infertility was not made to the participants and their views may have been different for the two forms of infertility. The knowledge assessed on the treatment options was limited to the advanced reproductive technologies and its awareness is expected to be low in the general public. As in other studies of this kind, convenience sampling was a limitation as the ideas gathered through this study may not represent views of the general population. Although minimized by training sessions and a structured interview, the interviewer bias may still have been present.

## Recommendations

Adequate knowledge is required so that infertile couples can seek medical care in a timely manner and prevalent myths and misconceptions can be rectified. Based on these findings, we recommend the knowledge regarding infertility in Pakistan to be made more readily available to the general population. This can be done through the media via numerous health shows, which are popular amongst people. Television will be the best way to target the less educated, who not only have a poor knowledge base but also are more willing to accept the alternative causes of infertility and more likely to seek alternative treatments.

Religious authorities should be involved in order to present the correct Islamic views available to the public. They can inform the public that Islam does allow people to seek medical help for infertility and permits IVF as a treatment option, as long as no third-party donation is involved. It is also important for the public to know that according to Islam, infertility is no grounds for divorce. It may also be beneficial to assess the understanding and beliefs of religious leaders about infertility. Assessing their knowledge may be the key to identifying the source of misinformation and reasons for low acceptability of ART in the society.

## Conclusion

Infertility is a fairly common problem affecting approximately one-fifth of the population. We discovered that the knowledge about infertility is generally limited amongst the participants. In fact, there are a lot of misconceptions, such as people's beliefs that IUCD and OCPs can cause infertility. People still believe in supernatural powers as a cause of infertility and seek treatment from faith healers. Alternative medicine is also a popular option for couples in case they are not satisfied with allopathic medicine. Knowledge about treatment options is also lacking and its cultural and religious perspective is unclear, which has resulted in reduced acceptability of assisted reproductive technologies.

## Abbreviations

(WHO): World Health Organization; (IVF): In-vitro Fertilization; (OCP): Oral Contraceptive Pills; (IUCD): Intrauterine Contraceptive Device; (ART): Assisted Reproductive Technologies.

## Competing interests

The authors declare that they have no competing interests.

## Authors' contributions

RS, SA, SFUH were involved in study conception and design. RS, SA, AMI, FIK and SFA were involved in data analysis. SA, RS, AMI, FIK, AS and SFUH contributed to the manuscript. SFUH provided overall supervision and guidance in the project. All authors read and approved the final manuscript.

## Pre-publication history

The pre-publication history for this paper can be accessed here:

http://www.biomedcentral.com/1471-2458/11/760/prepub
